# Hypopituitarism due to central nervous system germinoma: a case report

**DOI:** 10.1186/1687-9856-2015-S1-P118

**Published:** 2015-04-28

**Authors:** DoThi Thanh Mai, Vu Chi Dung, Bui Phuong Thao, Nguyen Ngoc Khanh, Can Thi Bich Ngoc, Nguyen Phu Dat

**Affiliations:** 1 National Hospital of Pediatrics, Hanoi, Vietnam

## 

Germ cell tumors in the central nervous system affect children and adults, the peak incidence is from 10 -19 years of age. Clinical presentation is mainly involving to the location and size of the tumor and the patient age. Endocrine abnormalities are the most common symptom.

We presented a case who had hypopituitarism due to central nervous system Germinoma.

A 10 years old girl was admitted to our hospital due to polyuria . Past history : she was diagnosed with diabetes insipidus and treated with oral minirin ( desmopressin ) 3 years ago. But she did not taken oral minirin for 2 years ago.

Physical examination showed : polyuria ( 8 ml/kg/h ), polydipsia, no dehydration, no weight gain. Her height was 131 cm ( -0.5 SD ). Her weight was 41 kg. Her BMI was 23.9 ( > 97th ). She had no manifestation of puberty. Laboratory evaluation revealed : blood osmotic pressure : 304 mosm/kg (normal range : 275 mosm/kg – 295 mosm/kg ), urinary osmotic pressure : 80 mosm/kg ( normal range : 500 – 1200 mosm/kg); serum cortisol at 8 a.m : 9.2 nmol/l ( normal range : 200 – 600 moll/l ); plasma glucose level and electrolyte were normal; T4 : 38.8 nmo/l ( normal : 50 -150 nmol/l ), T3 : 2.35 nmol/l (normal range : 1 – 3 nmol/l ), TSH : 4.1 mcUI/ml ( normai range : 1 – 5 mcUI/ml ); LH : 0.16 UI/ ml (normal range : 0,5 – 9,9 UI/l ),, FSH : 0.9 UI/ml (normal range : 1,4 – 5,6 UI/l), Estradiol < 43.3 pmol/l ( normal range : 97 – 169 pmol/l ). An MRI of brain showed: an under hypothalamic mass measuring 14 × 20 mm, suspected Germinoma.

She was treated with hormone replacement (desmopressin, levothyroxine, hydrocortisone) and radiation therapy.

Written informed consent was obtained from the patient's parent or guardian for publication of this Case report (and any accompanying images). A copy of the written consent is available for review by the Editor-in-Chief of this journal.

